# The Relationship Between SARS-CoV-2 Neutralizing Antibody Titers and Avidity in Plasma Collected From Convalescent Nonvaccinated and Vaccinated Blood Donors

**DOI:** 10.1093/infdis/jiad070

**Published:** 2023-03-27

**Authors:** Visa Nurmi, Chanice Knight, Lise Estcourt, Jussi Hepojoki, Abigail A Lamikanra, Hoi P Tsang, David J Roberts, Fernando P Polack, Peter Simmonds, Klaus Hedman, Damian Alvarez-Paggi, Heli Harvala

**Affiliations:** Nuffield Department of Medicine, University of Oxford, Oxford, United Kingdom; Department of Virology, Faculty of Medicine, University of Helsinki, Helsinki, Finland; Nuffield Department of Medicine, University of Oxford, Oxford, United Kingdom; Clinical Services, NHS Blood and Transplant, Oxford, United Kingdom; Radcliffe Department of Medicine and Biomedical Research Centre Haematology Theme, University of Oxford, Oxford, United Kingdom; Department of Virology, Faculty of Medicine, University of Helsinki, Helsinki, Finland; Institute of Veterinary Pathology, Vetsuisse Faculty, University of Zürich, Zürich, Switzerland; Clinical Services, NHS Blood and Transplant, Oxford, United Kingdom; Radcliffe Department of Medicine and Biomedical Research Centre Haematology Theme, University of Oxford, Oxford, United Kingdom; Clinical Services, NHS Blood and Transplant, Oxford, United Kingdom; Clinical Services, NHS Blood and Transplant, Oxford, United Kingdom; Radcliffe Department of Medicine and Biomedical Research Centre Haematology Theme, University of Oxford, Oxford, United Kingdom; Fundación Infant, Buenos Aires, Argentina; Nuffield Department of Medicine, University of Oxford, Oxford, United Kingdom; Department of Virology, Faculty of Medicine, University of Helsinki, Helsinki, Finland; Diagnostic Centre, Helsinki University Hospital, Helsinki, Finland; Fundación Infant, Buenos Aires, Argentina; Radcliffe Department of Medicine and Biomedical Research Centre Haematology Theme, University of Oxford, Oxford, United Kingdom; Microbiology Services, NHS Blood and Transplant, Colindale, United Kingdom; Infection and Immunity, University College of London, London, United Kingdom

**Keywords:** COVID-19, SARS-CoV-2, avidity, convalescent plasma, IgG, neutralizing antibody, treatment

## Abstract

Convalescent plasma (CP) treatment of coronavirus disease 2019 (COVID-19) has shown significant therapeutic effect when administered early (eg, Argentinian trial showing reduced hospitalization) but has in general been ineffective (eg, REMAP-CAP trial without improvement during hospitalization). To investigate whether the differences in CP used could explain the different outcomes, we compared neutralizing antibodies, anti-spike IgG, and avidity of CP used in the REMAP-CAP and Argentinian trials and in convalescent vaccinees. We found no difference between the trial plasmas, emphasizing initial patient serostatus as treatment efficacy predictor. By contrast, vaccinee CP showed significantly higher titers and avidity, being preferable for future CP treatment.

**Clinical Trials Registration**. NCT02735707 and NCT04479163.

## Teaser

Outcomes of 2 clinical SARS-CoV-2 convalescent plasma treatment trials, one effective the other ineffective, were not explained by serological markers of the convalescent plasma used. Start of treatment in relation to disease progression was likely the key factor that determined success.

Despite successful development of targeted antiviral therapy for severe acute respiratory syndrome coronavirus 2 (SARS-CoV-2), there is a need for more effective treatment options for severely affected individuals and better protection for vulnerable groups, such as the immunocompromised. While immune protection relies on both cellular and humoral immunity, the administration of SARS-CoV-2–specific neutralizing antibodies (nAb) can enhance host immunity. Convalescent plasma (CP) treatment using plasma collected from previously infected donors has been shown to have a significant therapeutic effect in terms of disease progression when administered early or before hospitalization [1, 2]. Multiple subsequent trials have observed no clinical benefit in terms of disease outcome or mortality when treating those more severely ill, unless they have impaired immunity [3–5]. Monoclonal anti–SARS-CoV-2 antibody therapy has shown to be effective, yet exclusively when given before antibody response [6].

As the clinical trials differ in patient demographics, treatment protocols, and timing in relation to the course of coronavirus disease 2019 (COVID-19), the reasons underlying the differences in efficacy of CP remain to be determined. Firstly, potential variability in the criteria used to select donors may influence CP potency. It is also possible that CP administration is only able to change the course of disease during very early stages, mimicking the role of vaccination, whereas this protective effect might be lost if administered after onset of the host's own response. In addition, assays used in CP characterization, which determine selection of high-titer antisera for transfusion, may vary between trials. High titers of nAb against SARS-CoV-2 are generally considered essential for protection, while some other antibody properties, such as afucosylation or association with antibody-dependent cellular cytotoxicity, have been considered potentially harmful [4]. Finally, no clinical trials to date have investigated the importance of immunoglobulin G (IgG) avidity, the average binding strength of a polyclonal antibody population towards an antigen, in therapeutic CP, although it is proposed to act as a favorable clinical outcome predictor in COVID-19 [7]. The wider picture is further complicated by emergence of new variants partly evading neutralization by antibodies raised against earlier variants or vaccines, potentially also affecting antibody avidity.

In this study, we have investigated whether the differences in nAb titers, spike protein binding, and avidity of plasma used in the Randomized Embedded Multifactorial Adaptive Platform for Community Acquired Pneumonia (REMAP-CAP) and Argentinian trials were associated with their markedly different clinical outcomes [1, 3]. We further compared these metrics in plasma collected from convalescent donors following vaccination to guide the future selection of potential donors of CP therapy.

## METHODS

### Convalescent Plasma Samples

The REMAP-CAP panel included 67 plasma samples collected during April to May 2020 from SARS-CoV-2–infected blood donors ≥28 days after resolution of their symptoms in England (ClinicalTrials.gov NCT02735707 [3]; Table 1). Of these, 56 had been used in the REMAP-CAP CP trial while 11 had been excluded due to low antibody levels (signal/cutoff ratio <6 in EUROimmun S-IgG assay).

**Table 1.** Comparison of REMAP-CAP and Argentinian Plasma and Trial as Well as Convalescent Plasma From Vaccinated Individuals

Characteristic	REMAP-CAP UK	Argentina	Vaccine	
			After 1st Dose	After 2nd Dose
Convalescent Plasma Trials				
Trial start-completion	9 Mar 2020–18 Jan 2021	4 Jun 2020–25 Oct 2020	…	…
Participants	Intensive care unit patients	Older adults with mild COVID-19 symptoms	…	…
No. of participants	2011	160	…	…
Average age, y	61	77	…	…
Male/female	68% / 32%	38% / 62%	…	…
Start of CP treatment	48 h after ICU admission (estimated 8 d after symptom onset)	Within 3 d (≤72 h) after symptom onset	…	…
Dosage, treatment/control	550 mL CP/no infusion	250 mL CP/250 mL saline	…	…
Primary end point, treatment/control	Organ support-free days, median (IQR), 0 (−1 to 16) / 3 (−1 to −16), death coded as −1	Severe respiratory disease, 16% / 31% Relative risk 0.52 (95% CI .29–.94)	…	…
Mortality rate, treatment/control	37% / 38%	2.5% / 5.0%	…	…
Convalescent plasma				
No. of donors	379	135	36	66
Donor plasma collection	22 Apr 2020–12 May 2020	4 Jun 2020–10 Oct 2020	26 Apr 2021–28 Jul 2021	28 Apr 2021–14 Aug 2021
Donor plasma inclusion cutoff	EUROimmun anti-spike IgG ratio ≥6	COVIDAR anti-spike IgG titer >1:1000	EUROimmun anti-spike IgG ratio ≥1, prevaccine	EUROimmun anti-spike IgG ratio ≥1, prevaccine
Time since SARS-CoV-2 infection	≥28 d after resolution of symptoms	≥3 d after resolution of symptoms + 2×negative PCR	≥92–396 d (median 202)	≥170–473 d (median 354)
Time since latest vaccine dose	…	…	33–79 d (median 55)	29–140 d (median 57)
Retrospective Convalescent Plasma testing				
No. tested	56	46	36	66
Mean IgG titer (range)	1952 (66–6492)	1147 (147–4945)	11 539 (1095–71 858)	6309 (1162–74 967)
Mean nAb titer (range)	120 (<20–1280)	98 (20–1280)	747 (160–≥5120)	472 (80–≥5120)
Mean IgG avidity (range)	0.25 (0.07–0.37)	0.19 (0.05–0.39)	0.58 (0.42–0.90)	0.59 (0.36–0.91)
Mean IgM titer (range)	332 (<100–3945)	255 (<100–2016)	127 (<100–3064)	109 (<100–458)

Abbreviations: CI, confidence interval; COVID-19, coronavirus disease 2019; CP, convalescent plasma; ICU, intensive care unit; Ig, immunoglobulin; IQR, interquartile range; nAb, neutralizing antibody; REMAP-CAP, Randomized Embedded Multifactorial Adaptive Platform for Community Acquired Pneumonia; SARS-CoV-2, severe acute respiratory syndrome coronavirus 2.

The Argentina panel included 61 plasma samples collected during June to October 2020 in Argentina ≥3 days after resolution of SARS-CoV-2 symptoms which had lasted ≥10 days; these donors also had 2 negative reverse transcription polymerase chain reaction (RT-PCR) results prior to donation (ClinicalTrials.gov NCT04479163 [1]; Table 1). From these, 46 donations had been supplied for the Argentinian CP trial while 15 had been excluded due to low antibody level (S-IgG titer ≤1000 in COVIDAR assay).

The vaccine panel included 102 plasma samples obtained during April to August 2021 from UK blood donors who had had a previous SARS-CoV-2 infection (range, 92–473 days prior to sampling, median 310 days, estimated based on the earliest seropositive prevaccine sample) followed by vaccination (range, 40–346 days before sampling, median 178 days [8]; Table 1). At the time of sampling, 36 had received one and 66 two doses of vaccine. The intervals between the vaccine doses varied from 21 to 95 days (median, 77 days).

### Ethics Statement

Signed consent was obtained from each donor at the time of donation. With UK donors, it included the use of data for the purpose of clinical audit to assess and improve the service provided by NHS Blood and Transplant as well as for research to improve our knowledge of the donor population. Approval for plasma samples collected from vaccinated donors was received from the West Midlands Solihull Research Ethics Committee, UK (REC-reference, 21/WM/0082; IRAS-project-ID, 296926). Approval for Argentinian plasma samples collected from convalescent donors was approved by Dirección de Sangre y Medicina Transfusional del Ministerio de Salud number, PAEPCC19, Plataforma de Registro Informatizado de Investigaciones en Salud number, 1421.

### SARS-CoV-2 Testing

**Figure 1.**
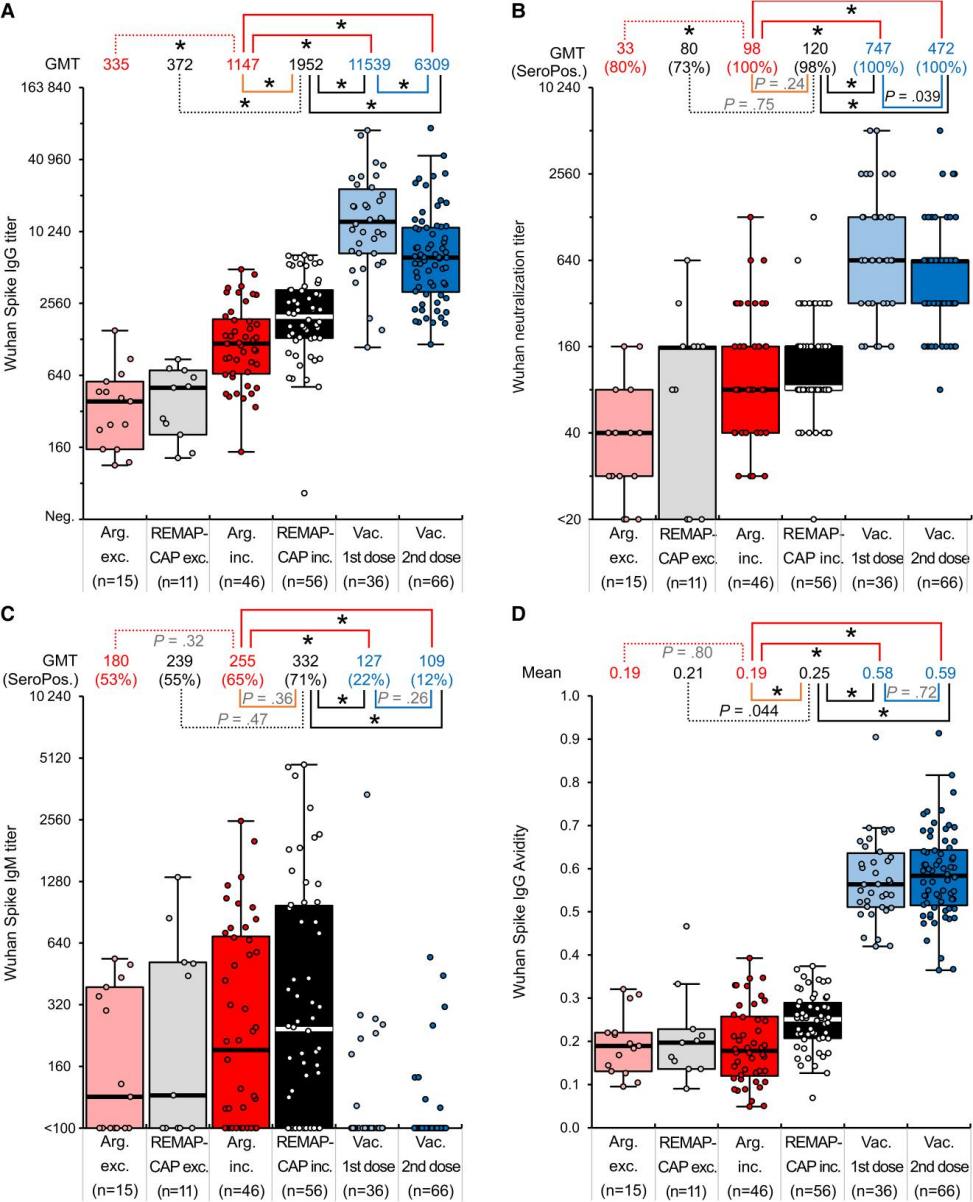
Comparison of SARS-CoV-2 Wuhan anti-spike IgG titers (*A*), neutralizing antibody titers (*B*), anti-spike IgM titers (*C*), and anti-spike IgG avidities (*D*) in REMAP-CAP donors (where plasma met criteria for inclusion, REMAP-CAP inc., or was excluded, REMAP-CAP exc.), Argentinian donors (where plasma met criteria for inclusion, Arg inc., or was excluded, Arg exc.) and vaccinated (after first and second dose) convalescent plasma. *P ≤ 1.8 × 10^−3^. Boxplots: Median, thick horizontal line; Wishkers, 1.5×interquartile range from 25th and 75th percentle (box); individual datapoints are overlayed. Abbreviations: GMT, geometric mean titer; Ig, immunoglobulin; Neg., negative; REMAP-CAP, Randomized Embedded Multifactorial Adaptive Platform for Community Acquired Pneumonia; SARS-CoV-2, severe acute respiratory syndrome coronavirus 2; SeroPos., seropositive.

Anti-Spike IgG titers were measured by enzyme-linked immunosorbent assay (ELISA) using spike antigen based on the first strains reported from Wuhan in January 2020 [9]. The samples were tested in 4-fold dilution series, and a titration curve was fitted to the series as previously described [10]. The titers were normalized against a calibrator plasma [11]. The ELISA was adapted for anti-spike IgG avidity: each sample was assayed in 2 dilution series; after antigen-binding, one series was incubated 3 times for 5 minutes with 4 M urea in phosphate-buffered saline Tween (PBST) and the other with PBST alone. Both series were washed once with PBST before applying anti-human IgG conjugate (Dako P0214); IgG avidity was calculated by the ratio of titers with and without urea, respectively, as described [10]. High avidity is indicative of stronger binding, reflecting higher antibody affinity. Anti-spike IgM was measured, as described for IgG, using peroxidase-conjugated goat anti-human IgM (Dako P0215; 1:1000 diluted). Neutralizing antibodies were detected using a live-virus microneutralization assay [12]. The nAb titer was determined as the concentration of serum that showed ≥50% virus neutralization as measured by cellular cytopathic effect. Avidities and log-transformed titers were analyzed in R version 4.1.2 software using Kruskal-Wallis (Figure 1*A*–1*D*), linear regression (Supplementary Figure 1*A*–1*C*), and Mann-Whitney-Wilcoxon (Supplementary Figure 1*D*) tests. Adjusted *R*^2^ values normalized by sample size are reported.

## RESULTS

Spike IgG levels and avidities as well as nAb titers were measured in plasma samples from the REMAP-CAP, Argentina, and vaccine panels.

### Comparison of Anti-Spike Reactivity and nAb Titers in Plasma Used in REMAP-CAP and Argentina Trials

We compared anti-spike IgG (S-IgG) reactivities of plasma included or excluded from trial use based on reactivity to spike protein with an in-house ELISA. For the REMAP-CAP panel, plasma included in the clinical trial showed a 5.2-fold higher geometric mean titer (GMT) of S-IgG than excluded plasma (GMTs of 1952 and 372, respectively; *P* = 4 × 10^−6^; Figure 1). Similarly, included and excluded plasmas in the Argentina trial showed a 3.4-fold difference in GMT (1147 and 335 respectively; *P* = 7 × 10^−6^). Higher mean nAb titers were similarly observed in plasma selected for clinical trial compared to plasma excluded for use; 3.0-fold higher GMT in Argentina panel (98 and 33, respectively; *P* = .0018) and 1.5-fold higher in REMAP-CAP panel (120 and 80, respectively; *P* = .75). Anti-spike IgM (S-IgM) titers did not differ significantly between REMAP-CAP and Argentina trials nor between plasma included or excluded from the trials (*P* = .32–.47). Furthermore, although the mean S-IgG titer of plasma samples used in the REMAP-CAP trial was 1.7-fold higher than the samples used in the Argentinian trial (*P* = 5 × 10^−4^), no difference in nAb titers was observed between the 2 sample sets (*P* = .24).

### Comparison of Antibody Avidity

We further compared antibody reactivities in the presence and absence of urea, which destabilizes antibody binding (see “Methods”). Measured avidities were similar between plasma excluded and included in the Argentinian trial (mean avidity 0.19; Figure 1*D*), and only marginally higher in plasma used in the REMAP-CAP trial (avidity 0.25). As with other metrics of serological reactivity, there were no significant differences in avidity between samples used in the Argentinian and REMAP-CAP trials.

### Effects of Prior SARS-CoV-2 Infection and Vaccination on Serological Reactivity

SARS-CoV-2 immunization led to an increase in serological reactivities, nAb titers, and avidity. S-IgG titers after first and second vaccination were 5.9- and 3.2-fold higher, respectively, than in plasma collected for the REMAP-CAP trial, and 10.1- and 5.3-fold higher than in Argentinian trial plasma (*P* < .05; Figure 1). Similarly, nAb titers were 6.2- and 3.9-fold higher compared to REMAP-CAP and 7.6- and 4.8-fold higher compared to Argentinian trial plasma (*P* < .05). Plasma collected from vaccinated convalescent donors also showed over 2-fold greater S-IgG avidity than plasma used in REMAP-CAP and Argentinian trials (mean 0.58 and 0.59 vs 0.19 and 0.25, respectively; *P* < .05). S-IgM seropositivity was 22% after the first vaccination and 12% after the second, compared with 71% and 65% in REMAP-CAP and Argentina trials, respectively.

### Interassay Correlations

There was a correlation between nAb and S-IgG titers (*R*^2^ = 0.72, *P* < 2 × 10^−16^; Supplementary Figure 1). Avidities correlated with estimated sampling times after primary infection, but with neither IgG titers (*R*^2^ = 0.03, *P* = .024; *R*^2^ = 0.03, *P* = .061) nor nAb titers (*R*^2^ = 0.03, *P* = .024; *R*^2^ = 0.07, *P* = .005) when analyzed within each distinct time point, that is shortly after primary infection (REMAP-CAP and Argentina) and several months later (vaccine panel), respectively. The ratio of nAb to S-IgG titer was, however, higher in vaccinated than in unvaccinated REMAP-CAP or Argentinian donors (*P* = 2 × 10^−5^).

## DISCUSSION

In this study, we have firstly compared the serological metrics of CP used in REMAP-CAP and Argentinian trials, considering their markedly different outcomes. Whereas the REMAP-CAP trial could not demonstrate effectiveness of CP when administered to severely ill COVID-19 patients [3], the Argentinian trial showed reduction in the COVID-19 progression among older adult patients that received CP within 3 days from the onset of mild COVID-19 symptoms [1]. We have further compared these metrics to those obtained in plasma collected from convalescent donors following vaccination.

As no differences in serological reactivity, nAb titers, or avidities in the plasma used in these 2 trials were observed in the present study (Figure 1), it appears likely that their therapeutic potencies were equivalent, whereby additional explanations such as timing or populations treated will more likely account for the different clinical outcomes. Aspects of patient selection may also have contributed to the trial outcomes; in the REMAP-CAP trial, 68% of patients were male, also shown to be at elevated risk of developing severe SARS-CoV-2 infection, whereas in the Argentinian trial men accounted for merely 38% of those treated.

In terms of antibody maturation (low avidity, IgM positivity) the plasma used in REMAP-CAP and Argentinian trials was typical for endogenous antibodies produced during primary SARS-CoV-2 infection. Antibody-mediated SARS-CoV-2 uptake by monocytes and macrophages has been suggested to trigger inflammatory cell death leading to systemic inflammation that further modulates COVID-19 pathogenesis, but such antibody-mediated enhancement is not seen with vaccinee plasma [13]. For these reasons, as well as their higher nAb titers and avidity, it might be beneficial to collect CP from vaccinated individuals to avoid pathology, especially if plasma was given to patients during late-stage SARS-CoV-2 infection, as demonstrated by the high pretreatment (70%) IgG seropositivity in the REMAP-CAP trial.

The therapeutic value of vaccine-derived CP is likely further enhanced by its previously described broadly neutralizing antibody response to new SARS-CoV-2 variants, including Omicron, which is important as such continue to emerge [8]. This issue was also considered at the time of REMAP-CAP trial analyses when the emergence of Alpha variant became obvious [11] and which led to a substantial loss of efficacy of most available monoclonal antibody therapies [14]. We should also aim to determine the neutralizing antibody threshold for effective CP treatment, similar to what has been previously determined for vaccine efficacy [15].

However, enrolment of convalescent blood donors is now more laborious than in the beginning of the pandemic as SARS-CoV-2 infections are not currently that well recorded. It is also currently unknown to what extent the initial exposure, whether infection or vaccine, defines the antibody response that is later enhanced by booster vaccinations or infections (the original antigenic sin). While the present vaccine immunity is mostly against the original Wuhan strain, use of plasma of recently infected donors could better account for SARS-CoV-2 evolution whereas monoclonal antibodies or vaccines may need constant updating.

In conclusion, we found no difference between CPs from REMAP-CAP or Argentina trials. As exemplified by the difference in the REMAP-CAP and Argentinian cohorts, simple antibody supplementation is clearly of greater value for patients prior to anti–SARS-CoV-2 seroconversion. Furthermore, plasma collected from convalescent donors following vaccination should be preferable for future CP treatment.

## Supplementary Data

Supplementary materials are available at *The Journal of Infectious Diseases* online. Consisting of data provided by the authors to benefit the reader, the posted materials are not copyedited and are the sole responsibility of the authors, so questions or comments should be addressed to the corresponding author.

## Supplementary Material

jiad070_Supplementary_Data

## Notes

***Acknowledgments.*** We thank all of those who contributed to organizing the vaccinated donor study at NHS Blood and Transplant including the Plasma for Medicine Programme, the Birmingham New Street Blood Donor Centre, and the NHSBT Clinical Trials Unit.

***Financial support*.** This work was supported by the European Union and European Blood Alliance: grant No. 101015756, project ‘SUPPORTing high-quality evaluation of COVID-19 convalescent plasma throughout Europe’ (SUPPORT-E) (to H. H., D. J. R., and L. E.); National Institute for Health and Care Research (NIHR): project ‘COVID-19-RECPLAS’ (to D. J. R. and L. E.); Agencia Nacional de Promoción de la Investigación, el Desarrollo Tecnológico y la Innovación: project ‘PICTO-COVIDINMUNIZACION00004’ (to D. A. P.); Academy of Finland (to J. H.); Finnish Society for Study of Infectious Diseases (to V. N.); NHS Blood and Transplant (to H. H., D. J. R., L. E., A. L., and H. P. T.); Sigrid Jusélius Foundation (to V. N. and K. H.); and Magnus Ehrnrooth Foundation (to V. N. and K. H.).
